# American

**Published:** 1896-08

**Authors:** 


THE AMERICAN VETERINARY COLLEGE, in its twenty-second annual announcement for the session of '96 and 97, retains its old faculty, to which has been added Professor Allen S. Heath, M.D., V.S., as Professor of Hygiene, Breeding, and Zoötechnics; Dr. Heath is well known to the veterinary profession and is a very well-informed man, and will contribute, we have no doubt, much valuable information to the students that may attend the college the coming session. Minor changes have been made in the hospital staff of graduates of the Class of '96; the session will open on September 30th and close with commencement exercises on March 25th. All matriculates at the school after January 1st will have to complete regents' examination or exhibit evidence of having collegiate education credentials to a certain point denominated in the announcement.

